# The effect of high-fat diet and exercise on KISS-1/GPR54 expression in testis of growing rats

**DOI:** 10.1186/s12986-020-00517-0

**Published:** 2021-01-06

**Authors:** Junpeng Feng, Rui Xu, Yafei Li, Qishu Zhou, Ge Song, Yimin Deng, Yi Yan

**Affiliations:** 1 Beijing Sports University, Beijing, 100084 China; 2 Nanjing Sport Institute, Nanjing, 210014 China

**Keywords:** Growing period, Rat, Testis, KISS-1/GPR54

## Abstract

**Purpose:**

To find the expression of KISS-1 and G protein-coupled receptor 54 in rats testis from PND 21st to 56th.

**Method:**

128 three-week-old weaned rats underwent high-fat diet and exercise (60–70% VO_2max_, 1 h/day, 5 days/week) intervention and were randomly divided into group C, CE, HC, or HE. Sample time points were set on the PND 21st, 35th, 43rd, and 56th. The testicular testosterone and the mRNA content, and protein content of KISS-1 and GPR54 in testis tissue were detected by ELISA, RT-qPCR, and Western blotting.

**Result:**

(1) The protein of KISS-1 and GPR54 increased gradually during the growing period. KISS-1 mRNA peaked at 35D and GPR54 peaked at 43D. (2) High-fat diet affected the expression of the KISS-1/GPR54 system in rat testis and reduced the expression level of KISS-1 protein. (3) 60–70% VO_2_max exercise decreased the KISS-1/GPR54 expression level. Exercise intervention improved testicular development in rats with a high-fat diet.

**Conclusion:**

The expression of KISS-1/GPR54 increased during the growing period. High-fat diet can downregulate the protein and gene expression of KISS-1/GPR54 and change the expression trend. 60–70% VO_2_max exercise decreased the expression of KISS-1/GPR54, which may be involved in the effects of exercise on high-fat dietary sex hormone disorders.

## Introduction

The discovery of the KISS-1/ G protein-coupled receptor 54, GPR 54 system has provided a new way to understand the link between energy and reproduction. In recent years, studies have shown that the axons of KISS-1 neurons attach to GnRH neurons and GPR54 neurons that are located on GnRH neurons. Intervention of KISS-1 or GPR54 gene expression can affect GnRH secretion, which supports the view that the KISS-1/GPR54 system directly regulates GnRH secretion by GnRH neurons and thus regulates gonadal development [1]. The KISS-1/GPR54 system is recognized for its ability to control multiple levels of the hypothalamic–pituitary–gonadal (HPG) axis, such as the onset of puberty, which can be regulated by exercise.

Our previous studies found that high-fat diet intervention could upregulate the expression of KISS-1/GPR54 in the hypothalamus of growing male rats, but it could also lead to lipid droplet deposition in testicular tissue and decrease the serum testosterone level.

According to its gene expression profile, KISS-1 is mostly highly expressed in the brain, followed by tissues such as placenta, testis, pancreas, liver, and small intestine; GPR54 is also highly expressed in the brain, followed by tissues such as pancreas, placenta, and testis [2]. However, the function of the KISS-1/GPR54 system in peripheral tissues is still unclear [3–7].

Recent data indicates that kisspeptin, encoded by the KISS1 gene, could play a role in transducing metabolic information into the HPG axis, the mechanism that controls reproductive functions [7, 8]. Severe obesity or a wasting state can affect normal puberty initiation and can lead to abnormal growth and development and gonadal disorders [9]. Previous studies have found that there is an imbalance between the androgen ratio in obese boys; hormone-induced male obese rats have disordered sex hormones, and the hypothalamic-pituitary–testicular axis structure shows pathological changes in gonadal development [9–11].

In recent years, exercise has attracted attention as an effective intervention to reduce energy accumulation, regulate energy imbalance, and reduce body fat to improve the adverse effects of a high-fat diet in various ways. Swimming, moderate-intensity treadmill exercise, wheel running, and other sport forms have been shown to improve rat fatness caused by a high-fat diet by increasing energy consumption, increasing white fat browning, and reducing fat accumulation [12, 13] For adverse effects on the development of the reproductive system, such as increased sperm apoptosis and enhanced oxidative stress in high-fat diet/obese rats, long-term regular exercise provides protection via antioxidant enzymes such as catalase, SOD, and glutathione peroxidase. Damage to testicular tissue spermatogenesis is caused by increased reactive oxygen species (ROS). Yi et al. explored the effects of high-fat diet and exercise on testosterone synthesis and the leptin pathway in testis of male C57BL/6J mice. After 10 weeks of high-fat intervention, high-intensity or moderate-intensity exercise intervention was carried out. They found that moderate and high-intensity exercise could significantly reduce body fat and increase gene and protein expression levels of the LEP-JAK-STAT pathway [12]. However, only moderate intensity exercise can significantly increase the gene and protein expression levels of StAR and P450scc related to testosterone synthesis, thereby improving sex hormone levels. Both animal and human experiments have shown that exercise can regulate obesity-induced sex hormone disorders, but its mechanism of action is still unclear.

In view of, (1) the KISS-1/GPR54 system being a puberty-initiated control switch; (2) the KISS-1/GPR54 system existing in the gonads, but with an unclear function; (3) Exercise being able to regulate obesity-induced sex hormone disorders, but with an unclear mechanism, we hypothesized that the KISS-1/GPR54 system in gonadal tissue is involved in the regulation of gonadal development during the growing period, and that the KISS-1/GPR54 system is involved in the regulation of sex hormone disorders induced by high-fat diet, and that exercise can regulate these disorders. We studied the changing KISS-1/GPR54 system in testis during the growing period, and then based on this, we explored the possible relationship between KISS-1/GPR54 with testicular tissue testosterone.

## Materials and methods

### Animals

All experiments were approved by the Department of Zoology Research Committee, Beijing Sport University. 128 three-week-old weaning SD rats were purchased from Beijing Weitonglihua Laboratory Animal Co. Ltd., Beijing (License No. SCXK Beijing 2012-0001). Animals were housed under standard laboratory conditions (light:dark 12 h:12 h) and were provided with pelleted food and water ad libitum.

Animals were randomly divided into standard diet group (group C, n = 32), standard exercise group (group CE, n = 32), high fat diet group (group HC, n = 32) and high fat exercise group (group HE, n = 32). Groups C and CE were fed with control feed(3.82 kJ g^−1^; 10% energy from fat, 20% from protein and 70% from carbohydrate; Beijing Huafukang Biotechnology Co. Ltd., LOT No: D12451B); groups HC and HE were fed with high-fat feed with 45 kcal% Fat (4.7 kJ g^−1^; 45% energy from fat, 20% from protein and 35% from carbohydrate; Beijing Huafukang Biotechnology Co. Ltd.,LOT No: D12451),using the same formulation with D12451 from Research Diets, USA.

### Endurance exercise training

Group CE and HE rats were habituated to treadmill exercise, during which each rat walked on a motor-driven treadmill at 10 m/min (0-degree incline), and during the first three days, the durations were 10 min, 20 min, and 30 min. After the habituation period, the incline was raised to 10-degrees for the duration of the training period. Training included warm-up (5 min), training (50 min), and recovery (5 min). The running speeds of warm-up and recovery stages were 10 m/min; the running speed of the training stage was 60–70% vVO2 max, with training 5 days per week. The specific training program was as follows:

### Sample collection

The rats of each group were classified into following age groups: (a) PND 21st day (21D, early childhood); (b) PND 35th day (35D, pre-puberty), (c) PND 43rd day (43D, puberty), (d) PND 56th day (56D, maturity). For the impact of acute exercise, each sample collection was taken at 48 h after the end of the last endurance exercise training. The body mass of each mouse was recorded before killing; 2% sodium pentobarbital was used to anesthetize them by intraperitoneal injection at a dose of 0.25 ml/100 g body weight. After anesthesia, the testes on both sides were taken, the testicular fat and epididymis were removed, and the mass of the two testes was determined. Testis were kept at −80 °C for RT-qPCR and Western blotting.

### RT-qPCR gene expression analysis

Total RNA was isolated from testes using an RNAprep Pure Tissue Kit (Cat. No: DP431, TIANGEN BIOTECH BEIJING CO., LTD.) following the manufacturer’s protocol. The time of the on-column DNA digestion was extended from 15 to 45 min to ensure complete removal of genomic DNA. The RNA was reverse transcribed into cDNA using TOYOBO ReverTra Ace qPCR RT Kit (Cat. No: FSQ-101, TOYOBO., LTD. LIFE SCIENCE DEPARTMENT) following the protocol provided by the manufacturer. The primer sequences were: KISS1 (Forward: ATGATCTGCTGGCTTCTTG; Reverse: CTGTGGGTTCAGGGTTCAC), GPR54 (Forward: CCACATGTGCCACTTTGACA; Reverse: AACCCACCAGATGCTAAGG), 18S (Forward: CTA GCACCATGAAGATCAAGATC; Reverse: ACTCATCGTACTCCTGCTTGC). After the two-step PCR reaction procedure, the amplification curve and the melting curve of real-time PCR were confirmed, a standard curve was prepared for PCR quantification, and the gene expression amount of each sample was obtained by CT value.

### Western blotting protein expression analysis

Total protein was extracted from testis using PBS (Thermo Fisher, 10010023). Protein concentrations were determined using an assay kit (Thermo Scientific, BCA Protein Assay Kit). Then, 50 μg of protein lysates were loaded, separated by denaturing sodium dodecyl sulfate–polyacrylamide gel electrophoresis, and transferred to a polyvinylidene difluoride membrane (Millipore, Billerica). Membranes were incubated in blocking buffer (Tris-buffered saline containing 5% skim milk) for 1 h at 37 °C, followed by hybridization with anti-KISS-1 (1:2000 dilution, Abcam, ab19028) or anti-GPR54 (1:1000 dilution, Bioss, bs-2501R) primary antibody at 4 °C overnight. Then, membranes were hybridized with a horseradish peroxidase-conjugated rabbit immunoglobulin G secondary antibody (1:20,000 dilution, Abcam, ab6721) for 1 h. Protein bands were detected by chemiluminescence using a Western blotting luminol reagent (SUDGEN). Films were scanned using a Bio-Rad imager and the protein level was semi-quantified using Quantity One 1D image analysis software Bio-Rad 5.2.

### Statistical Analyses

The densitometric data are presented as the mean ± SEM. The bands obtained from immunoblots were normalized to GAPDH (1:10,000 dilution, Abcam, ab8245). All data were tested by a normality test and homogeneity of variance before analysis. After meeting the criteria, the results were statistically analyzed by multivariate analysis of variance. The three-factor analysis of variance was performed by setting the age of the rats (21D, 35D, 43D, 56D), dietary intervention factors (standard diet, high-fat diet), exercise intervention (with or without exercise) as the three factors. If there was a three-factor interaction between the independent variables, a separate effect test was used; if there was no three-factor interaction between the independent variables, then a two-factor interaction analysis was performed, and if there was a two-factor interaction, a separate effect test was performed, taking *p* < 0.05 as statistically significant and *p* < 0.01 as statistically significant.

## Results

### Changes in testicular mass and gonadosomatic index (GSI) during different stages of rat growth

**Fig. 1:**
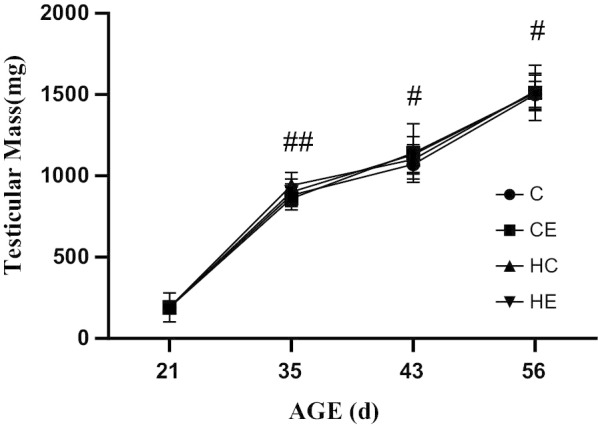
Testicular Mass of Growing Rats. *p < 0.05, **p < 0.01 when compared with the same sample point C Group; &*p* < 0.05, &&*p* < 0.01 when compared with the same sample point CE Group; $*p* < 0.05, $$*p* < 0.01 when compared with the same sample point HC Group; ^#^*p* < 0.05, ^##^*p* < 0.01 when compared with the same group at last sample point

**Fig. 2:**
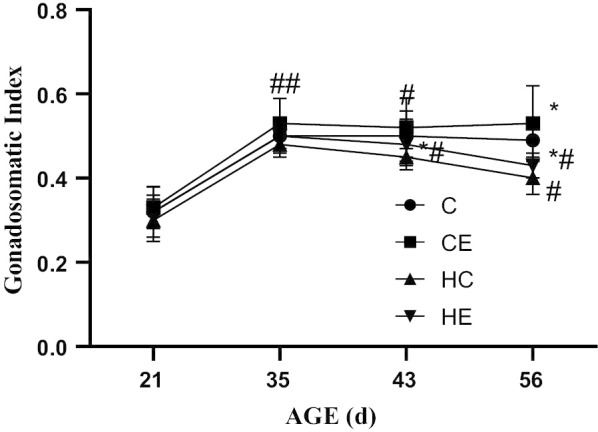
The GSI of growing rats

The results showed that both the testicular mass and GSI increased gradually from early childhood to maturity (Figs. 1 and 2). Exercise intervention or high-fat diet intervention had no effect on the testicular mass (*p* < 0.05). The high-fat diet intervention significantly reduced rat GSI, while exercise intervention significantly increased rat GSI, showing that exercise intervention can improve the GSI of high-fat diet rats. The body weight of growing rats as Additional file 1: Table S1 showed.

### Changes in testicular tissue testosterone during different stages of rat growth

**Fig. 3:**
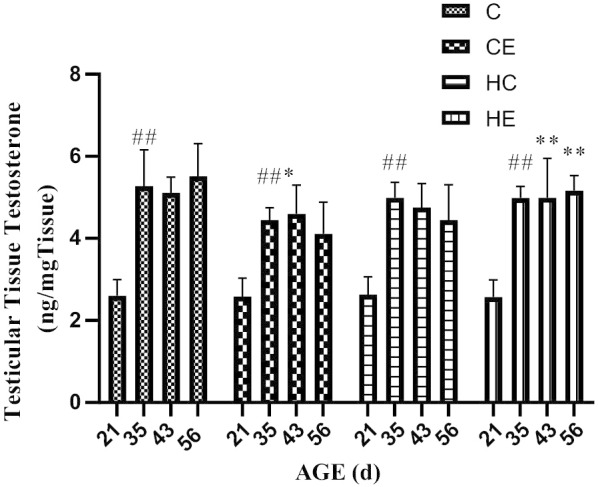
Changes in testicular tissue testosterone during different stages of rat growth

Figure 3 showed that testicular tissue testosterone increased significantly from PND 21st day-PND 35th day and became subsequently stable. Exercise intervention significantly downregulated testicular tissue testosterone levels in standard diet rats (*p* < 0.05). High-fat diet intervention also downregulated testicular tissue testosterone levels in rats (*p* < 0.01). Exercise intervention can improve testicular tissue testosterone levels in high-fat diet rats (*p* < 0.05).

### Changes in KISS-1/GPR54 expressions in the testis during different stages of rat growth

**Fig. 4:**
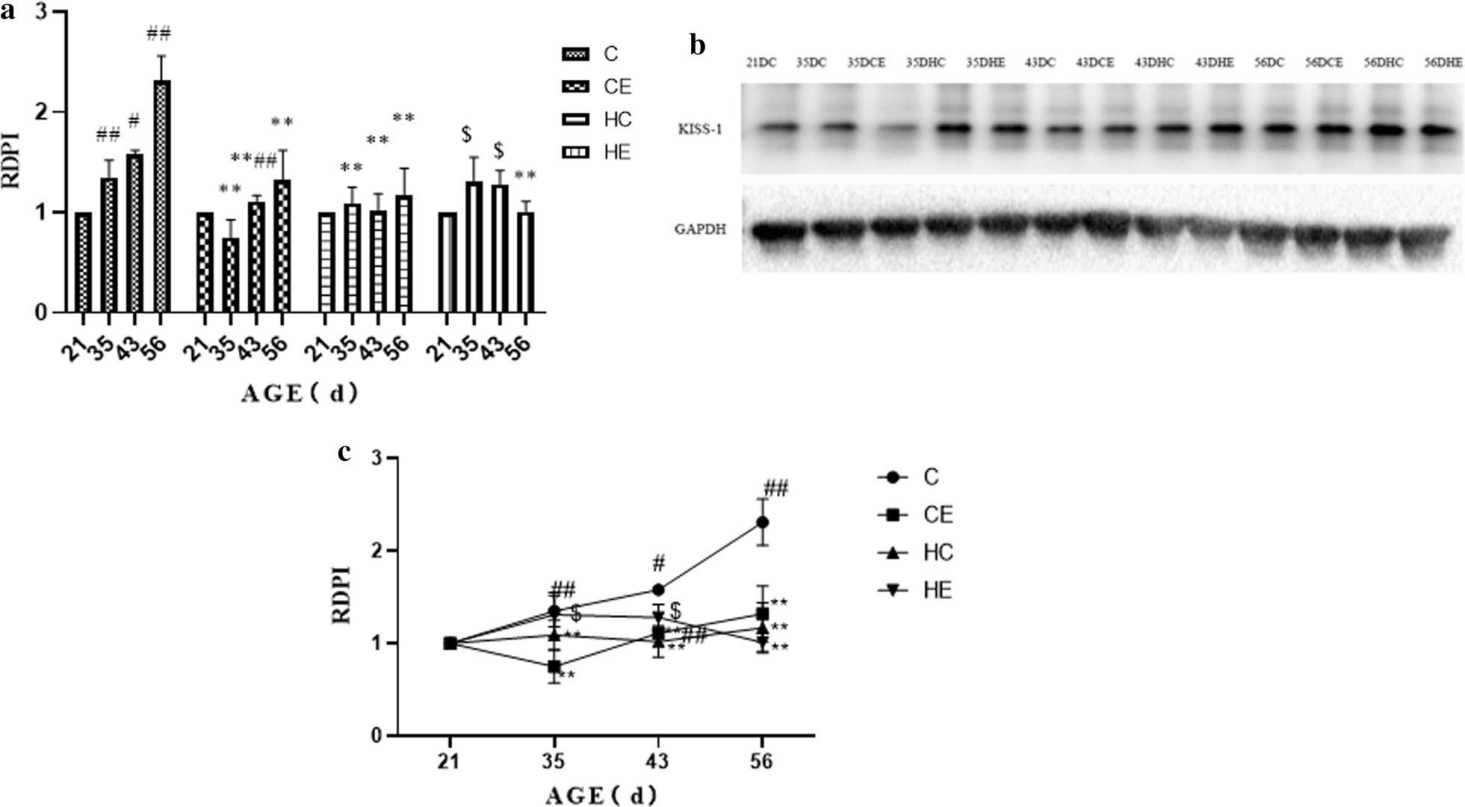
KISS-1 protein expression in the testis of different stages of rat growth. (as shown in Fig. 4a, c, KiSS-1 protein increases continuously in puberty and reaches its peak at maturity; exercise reduces KISS-1 protein expression level; high-fat diet leads to decrease of KISS-1 protein peak level; exercise can partially improve the abnormal expression of KiSS-1 caused by high-fat diet.)

**Fig. 5:**
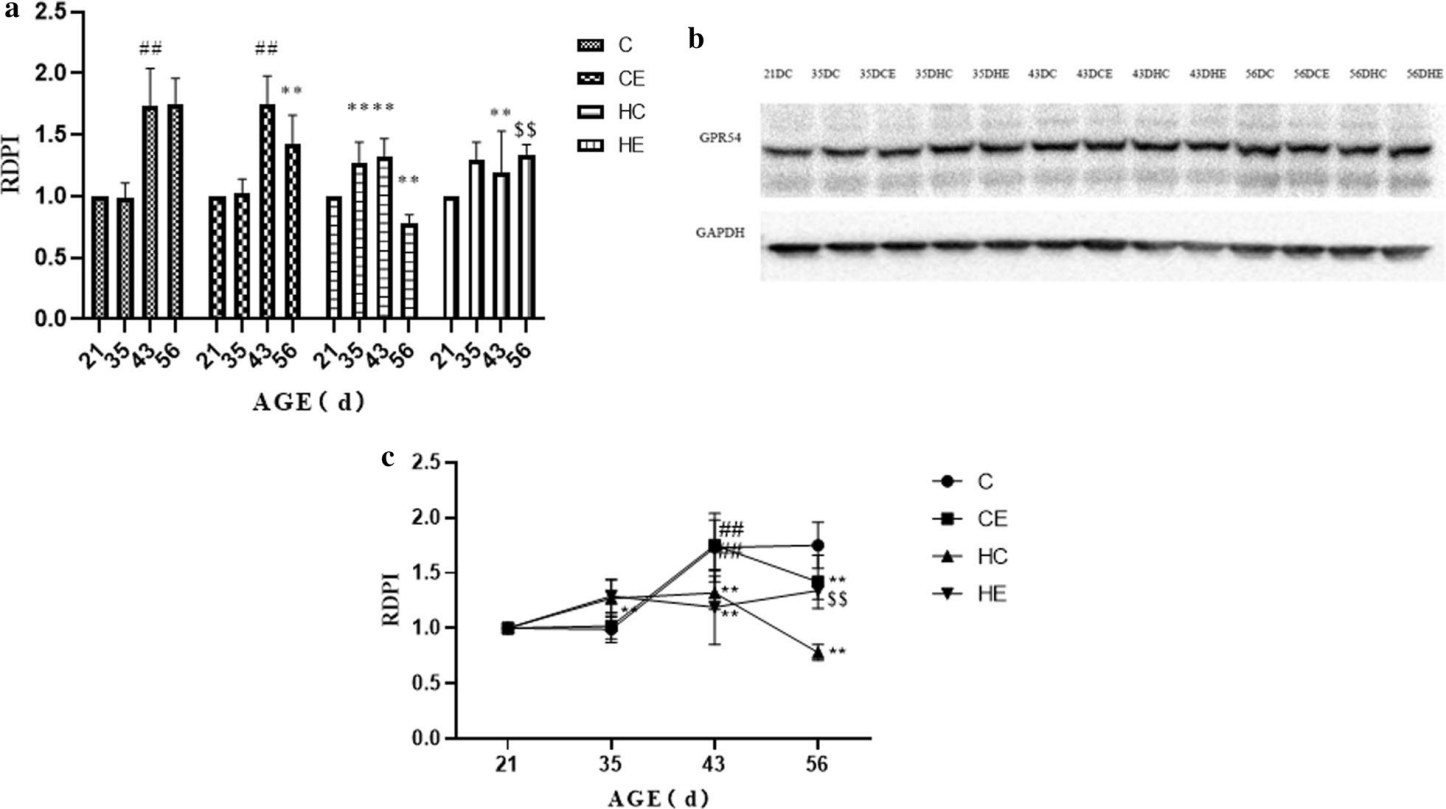
GPR54 Protein Expression in the Testis of Different Stages of Rat Growth. (As shown in Fig. 5a, c, GPR54 protein increased abruptly in puberty and reached maturity level; exercise did not change the expression trend of GPR54; high-fat diet led to the sudden increase of GPR54 protein at pre- puberty, and the peak level decreased; exercise can partially improve the abnormal expression of GPR54 induced by high-fat diet.)

Additional file 2 showed the immunolocalization of KISS-1 in rat testis. The densitometric analysis of testicular KISS-1 and GPR54 immunoblots from early childhood to maturity showed significant variation (Fig. 4). From early childhood to maturity, the immunoreactivity of KISS-1 increased significantly (*p* < 0.05). From early childhood to maturity, exercise intervention significantly reduced kisspeptin expression. From early childhood to maturity, high-fat diet intervention significantly reduced kisspeptin expression. Exercise intervention upregulated the decrease of kisspeptin expression caused by the high-fat diet, but the effect at maturity was not effective. As for GPR54, there was no significant change from early childhood to pre-puberty; then GPR54 expression increased significantly from pre-puberty to puberty, and from puberty to maturity the level remained relatively stable. Exercise intervention had no significant effect on the expression of GPR54 from early childhood to puberty, and then significantly downregulated the expression level of GPR54 in maturity. High-fat diet intervention upregulated the expression of GPR54 from early childhood to pre-puberty and significantly downregulated the expression level of GPR54 during maturity (Fig. 5).

**Fig. 6:**
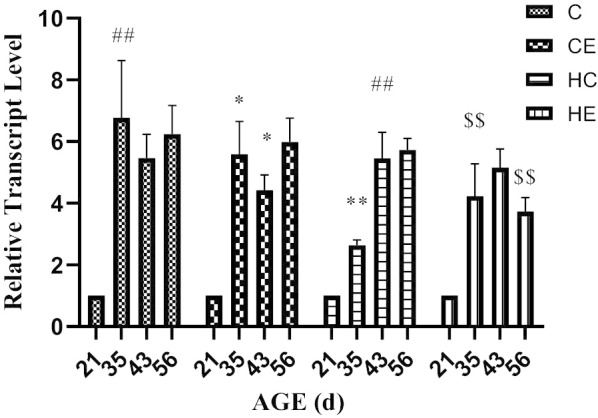
KISS-1 mRNA expression in the testis of different stages of rat growth

**Fig. 7:**
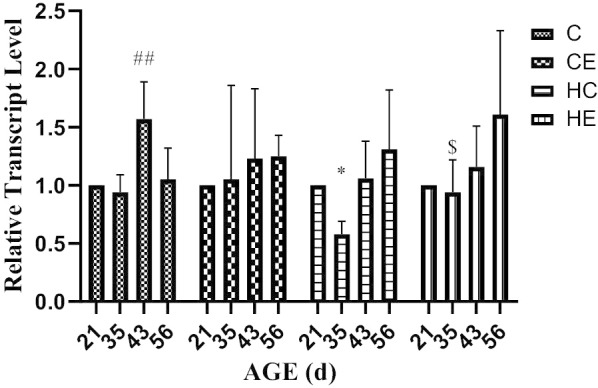
GPR54 mRNA expression in the testis of different stages of rats growth

Figure 6 showed that the expression of KISS-1 mRNA in rat testis increased significantly at 21D–35 D, and then stabilized (*p* < 0.01). Exercise intervention did not change the KISS-1 mRNA expression trend in rat testis, but decreased the expression level, especially in pre-puberty and puberty (*p* < 0.05). High-fat diet intervention significantly reduced the expression of KISS-1 mRNA in pre-puberty (*p* < 0.01), and then the expression of KISS-1 mRNA increased, but with no significant difference with the C group. Exercise intervention for rats fed with a high-fat diet changed the expression trend of KISS-1 mRNA. The expression trends of GPR54 mRNA and GPR54 protein were similar; both increased significantly from pre-puberty and puberty, and remained stable for the rest of the time (Table 1). Exercise intervention downregulated the expression level of GPR54 mRNA in adolescence, and for the rest of the time period, it was not significantly different from that in group C. High-fat diet intervention significantly reduced the expression of GPR54 mRNA in pre-puberty and puberty (*p* < 0.01). Exercise intervention improved the expression of GPR54 mRNA caused by a high-fat diet, but there was no significant difference (Fig. 7).

**Table 1:** Exercise intervention program

Group	Intensity (%vVO_2max_)	Incline (°)	Duration (min)	SPEED (m/min)				
				4 W	5 W	6 W	7 W	8 W
CE	60–70	10	60	12	14	15	16	17
HE	60–70	10	60	12	13	14	15	16

### Regression analysis of testicular development, KISS-1/GPR54 expression, and testicular tissue testosterone in rats

Regression analysis of GSI and testosterone concentrations in testicular tissues of rats at different stages and with different intervention factors was conducted. Table 2 showed that there was a significant correlation between KISS-1/GPR54 expression and testosterone concentrations in testicular tissues of rats only at a few special times (*p* < 0.05).

**Table 2:** Regression analysis of testicular development and testicular tissue testosterone in rats

	Result			
	F	P	R^2^	β
HE 43D GSI	25.975	0.007	0.833	−0.931
HC 56D GSI	12.691	0.024	0.700	0.872

Regression analysis of KISS-1/GPR54 system gene and protein expression levels in testicular tissues, and testicular tissue testosterone concentrations in different stages and with various intervention factors. A correlation existed only at a few special times between KISS-1/GPR54 expression in testis and testicular tissue testosterone concentration (*p* < 0.05), as Table 3 showed.

**Table 3:** Regression analysis of KISS-1/GPR54 expression and testicular tissue testosterone in rats

	Result						
	F		P		R^2^		β
C 56DKISS-1 Protein	14.240		0.020		0.726		0.884
CE 35DKISS-1 Protein	12.269		0.025		0.693		0.868
HC 56DGPR54 Protein	7.589		0.049		0.569		0.809

## Discussion

### Changes of KISS-1/GPR54 in testis of growing rats

In this study, the expression of the KISS-1/GPR54 system in testicular tissue of growing rats was studied at four time points: (a) PND 21st day (21 D, early childhood); (b) PND 35th day (35 D, pre-puberty), (c) PND 43rd day (43 D, puberty), and (d) PND 56th day (56 D, maturity). The results showed that from early childhood to early adolescence, the gonads of male rats developed rapidly. The GSI and testicular tissue testosterone increased significantly; gonad development slowed down from pre-puberty and puberty, then accelerated again from puberty to maturity, and finally developed completely. With gonadal development, KISS-1 was expressed in spermatogenic cells and interstitial cells of rat testis, and the expression of KISS-1 in interstitial cells increased significantly. GPR54 first appeared in spermatogenic cells, then in testicular interstitial cells. The protein expression of KISS-1 and GPR54 in testicular tissues increased gradually. KISS-1 protein increased rapidly from puberty to maturity, and GPR54 protein increased rapidly from pre-puberty to puberty. The expression level of KISS-1 first increased and then stabilized to a peak at PND 35th day, while the expression level of GPR54 first increased and then decreased, and peaked at PND 43rd day.

In this study, immunohistochemical results showed that the KISS-1/GPR54 system was expressed in spermatogenic cells and stromal cells in early childhood (21 D), but less in stromal cells, with low levels of protein and gene expression and low levels of testosterone. At this stage, the testicular supporting cells are still immature. At this time, the ability of phagocytosis is strong, which will inhibit the differentiation of spermatogonia. Also at this time, mesenchymal cells are at a low stage of development, and the number of mesenchymal cells in the field of vision is small. Anjum et al. found that kisspeptin was expressed in testes of newborn male Parkes mice, and the expression level decreased significantly from newborns (1 day old) to 4-weeks old (*p* < 0.05), mainly in interstitial cells [14]. The expression then decreased. Combined with gonadal development characteristics, we can find that early childhood is the low stage of development of rat testicular Leydig cells. This stage is mainly when testicular parenchyma develops, which suggests that the main function of the KISS-1/GPR54 system in early testis development may be related to the development of gonadal parenchyma.

In recent years, KISS-1 has been identified as an upregulated gene of the HPG axis, and its role in mammalian reproduction has been extensively studied [15, 16]. Ricu et al. showed that direct injection of kisspeptin antagonists into the ovaries before puberty resulted in delayed vaginal opening in female rats, suggesting that kisspeptin is directly involved in puberty initiation in females in the ovary [17]. In this study, the expression of kisspeptin in prepubertal testis increased in interstitial cells in addition to continued expression in spermatogonia, indicating that the KISS-1/GPR54 system in the testis continues to participate in testicular parenchymal development at this stage. It also may begin to participate in the secretion process of sex hormones.

With the end of puberty, the spurt of testicular gonadal development in rats ends, at which time the testicular tissue is mainly responsible for maintaining androgen secretion. For mature rats, related studies show that KISS-1 mRNA is mainly expressed in Leydig cells in adult rats, and that kisspeptin is mainly concentrated in Leydig cells during senescence with its expression level being correlated with the expression level of GnRH in testis. Our study also found that at PND 56, the expression level of KISS-1 protein in rat testis was significantly correlated with the testosterone concentration in testis, indicating that the main function of KISS-1 may be to regulate sex hormone levels when the rat enters maturity.

### Effect of high-fat diet on the expression of KISS-1/GPR54 in testis of growing rats

Here, we also studied the influence of high-fat diet (45%) intervention on the expression of the KISS-1/GPR54 system in testis tissue of rats in the growth phase. Our study found that high-fat diet intervention resulted in a decrease in the testicular index from pre-puberty in rats; testicular tissue testosterone decreased significantly from prepuberty, and endocrine function was impaired; rat testicular tissue structure was loose, and the number of mature sperm decreased. The high-fat diet (45%) intervention had no significant effect on the localization of KISS-1/GPR54 in rat testis, but affected its expression in rat testis by downregulating the expression of KISS-1 protein.

Related studies have shown that energy status and energy sources can affect pubertal initiation and gonadal development [18–21]. Cui et al. also showed that overnutrition from the lactation period caused the female vaginal opening time to advance from 39.10 ± 1.66 PND under normal conditions to 29.60 ± 1.96 PND, and the excess nutrients from the post-drug period caused vaginal opening time to advance to 35.80 ± 3.19 PND, so the earlier the timing of overnutrition, the greater the impact on female sexual development [22]. This study also showed the effect of dietary intervention on the KISS-1/GPR54 system in the hypothalamus, finding that excess energy caused the expression of KISS-1 in the hypothalamus of females at the PND 35 time point to be significantly higher than that of normal-diet females in PND 42. This stage was significantly lower than in the normal diet group, and the timing was earlier than the increase in GnRH.

As mentioned above, energy status can affect puberty initiation [21, 23, 24]. In view of the impact of high-fat diet on puberty development, especially reproductive system development, in recent years various forms of exercise to improve the adverse effects of high-fat diet have attracted attention. In this study, we explored the effects of exercise on KISS-1/GPR54 expression in testis of growing rats through moderate-intensity treadmill exercise intervention. The results showed that: (1) exercise intervention under standard diet could significantly increase testicular index and decrease testosterone concentrations in testicular tissue, but had no significant effect on the expression and localization of KISS-1/GPR54 in testis. Reducing KISS-1 protein and gene expression levels had no significant effect on GPR54 protein and gene expression; (2) Exercise intervention could increase the testicular index of rats fed with high-fat diet at different stages and improve testicular development inhibition caused by a high-fat diet; testosterone in testicular tissue increased gradually at each stage, and testosterone in testicular tissue was subtestosterone induced by the high-fat diet.

### Effect of exercise on the expression of KISS-1/GPR54 in testis of growing rats

The energy state of the body affects the expression of KISS-1/GPR54. The negative energy balance pattern of exercise, cold exposure, and fasting is mainly concentrated in the hypothalamus for the expression of KISS-1/GPR54, and there are also inconsistent results. In energy-deficient rats and sheep, hypothalamic KISS-1 mRNA is decreased, LH levels decrease, and puberty stops, but puberty returns to normal after the injection of kisspeptin, LH and androgen levels also return to normal. Similarly for the hypothalamus, if the KISS-1 mRNA expression between AVPV and ARC is studied separately, different results will be found. Yamada et al. found that the level of KISS-1 in ARC was decreased by lactation stimulation in lactating rats [25]. These studies suggest that the KISS-1 system in ARC is related to GnRH pulse generation and influenced by external factors. Kisspeptin neurons in AVPV are thought to mediate the positive feedback effect of estrogen on GnRH release, thus inducing the surge of GnRH. However, Byrne et al. found that 48-h fasting reduced the level of KISS-1 in AVPV, but not in ARC, and intravenous ghrelin also decreased the level of KISS-1 in AVPV. Currently, there are few studies on the effects of exercise on the KISS-1/GPR54 system in gonads.

Our study found that exercise under standard diet did not alter the expression of KISS-1 in rat testis tissue but downregulated KISS-1 protein and gene expression levels in rat testis; exercise did not alter the expression trend of GPR54 gene and protein from early childhood to adolescence, but led to the decreased expression of GPR54 protein at maturity. Correlation analysis found that at the PND 35th day, the testicular tissue testosterone decline caused by exercise was significantly correlated with the KISS-1 protein expression level, indicating the involvement of KISS-1/GPR54 in prepubertal testis. When the system begins to participate in the secretion of sex hormones, the lack of energy due to exercise may affect the level of sex hormones mainly by affecting the expression level of KISS-1.

On the other hand, this study found that exercise intervention can improve testicular development in rats with a high-fat diet, and can especially improve sex hormone disorders caused by high-fat diet. Under exercise intervention, the testicular tissue testosterone decline caused by a high-fat diet was improved. At the same time, the pre-puberty KISS-1/GPR54 system began to participate in the stage of sex hormone secretion, and exercise intervention upregulated the expression level of KISS-1/GPR54 at this stage. The specific mechanism leading to the downregulation of KISS-1 protein expression and gene expression after exercise intervention at maturity remains to be further studied.

### Possible mechanisms between KISS-1/GPR54 and testosterone secretion in testis

According to the results of the regression analysis and the changes in the expression of KISS-1/GPR54 in the testis, we found that the expression of kisspeptin in prepubertal testis increased in interstitial cells in addition to continued expression in spermatogonia, indicating that the KISS-1/GPR54 system in the testis continues to participate in testicular parenchymal development at this stage. It also may begin to participate in the secretion process of sex hormones.

As mentioned above, kisspeptin may participate in puberty initiation in the ovary [5, 26, 27]. Zhou et al. provided a high-fat diet and a standard diet to female SD rats after weaning and recorded the time of vaginal opening; 2 weeks after estrus and at 8–9 weeks of age, they found that the high-fat diet caused a significant increase in body weight in female rats, and puberty was advanced [28]. The high-fat diet disrupted the periodicity of estrus, and the expression of ovulation-related genes was significantly reduced. A previous KISS-1 study found that KISS-1 mRNA levels were significantly lower in high-fat diet rats at 42D estrus and in the 70D ovaries, and the expression of kisspeptin was significantly lower than in the standard group, suggesting the involvement of the ovaries. The KISS-1 system is downregulated under obese conditions and may be associated with related reproductive problems, such as ovulation dysfunction [29].

Our study found that the high-fat diet caused both KISS-1 protein and gene expression levels in testis to be significantly lower than in the standard diet group, significantly downregulated the expression of GPR54 at maturity, and changed the KISS-1/GPR54 system expression trend. At the same time, correlation analysis showed that testosterone levels in rat testis were significantly correlated with GPR54 protein expression at 56D, indicating that the decrease of GPR54 protein expression induced by a high-fat diet may be the influencing factor of testosterone decrease in rat testis.

In this study, we found that exercise intervention can improve testicular development under high-fat diet intervention by improving testicular tissue lipid droplet deposition and testicular tissue testosterone by means of morphology, testicular index, testicular tissue testosterone, and other indicators. The KISS-1/GPR54 system may be involved in improving androgen secretion, but the mechanism needs further study.

## Conclusion

1. The KISS-1/GPR54 system is expressed in rat testis during early childhood (21 D), prepuberty (35 D), puberty (43 D), and maturity (56 D). From early childhood to maturity, the expression of the KISS-1/GPR54 system increased and peaked at maturity (56 D).
2. A high-fat diet can inhibit the testicular development of male rats in the growth phase, can downregulate the protein and gene expression levels of the KISS-1/GPR54 system in testis tissue, and can change the expression trend of the KISS-1/GPR54. Its role needs further study.
3. 60–70% VO_2_max moderate-intensity aerobic exercise will downregulate the expression level of testis tissue KISS-1/GPR54, change the inhibitory effect of a high-fat diet on testicular development in male rats, and upregulate KISS-1/GPR54 in the prepubertal stage. Whether KISS-1/GPR54 in testicular tissue participates in its regulation requires further study.

## Supplementary information

**Additional file 1.** Supplement 1 Body Weight of Growing Rats.

**Additional file 2.** Supplement 2 Immunolocalization of KISS-1 in Rat Testis During Different Stages of Growing Rat (40X).

## Data Availability

The datasets used and/or analysed during the current study are available from the corresponding author on reasonable request.

## Abbreviations

ARC: Arcuate nucleus
AVPV: Antero ventral periventricular nucleus
GnRH: Gonadotropin-releasing hormone
GPR54: G protein-coupled receptor 54
GSI: Gonadosomatic Index
HPG: Hypothalamic–pituitary–gonadal
LH: Luteinizing hormone
PND: Postnatal day
