# Supplementary material for: The effect of high-fat diet and exercise on KISS-1/GPR54 expression in testis of growing rats
Source: Nutr Metab (Lond). 2021 Jan 6;18:1. doi: 10.1186/s12986-020-00517-0 (PMC7788936; doi:10.1186/s12986-020-00517-0)
Supplement: Supplementary file 1 — Additional file 1. Supplement 1 Body Weight of Growing Rats. [file 12986_2020_517_MOESM1_ESM.docx]

Supplement 1 Body Weight of Growing Rats

| GROUP | 21D | 35D | 43D | 56D |
| --- | --- | --- | --- | --- |
| C | 58.54±1.49 | 176.10±18.19## | 211.87±14.74# | 308.96±28.38# |
| CE | 58.41±1.53 | 163.28±14.73## | 215.58±14.59# | 264.13±25.91#* |
| HC | 58.45±1.69 | 193.82±11.94*## | 246.96±20.81#* | 324.52±37.52# |
| HE | 58.33±2.04 | 180.50±10.49&## | 233.70±16.15#& | 300.83±21.06#& |
