# Supplementary material for: The effect of high-fat diet and exercise on KISS-1/GPR54 expression in testis of growing rats
Source: Nutr Metab (Lond). 2021 Jan 6;18:1. doi: 10.1186/s12986-020-00517-0 (PMC7788936; doi:10.1186/s12986-020-00517-0)
Supplement: Supplementary file 2 — Additional file 2. Supplement 2 Immunolocalization of KISS-1 in Rat Testis During Different Stages of Growing Rat (40X). [file 12986_2020_517_MOESM2_ESM.docx]

Supplement 2 Immunolocalization of KISS-1 in Rat Testis During Different Stages of Growing Rat (40×)

|  | 21D | 35D | 43D | 56D |
| --- | --- | --- | --- | --- |
| C | 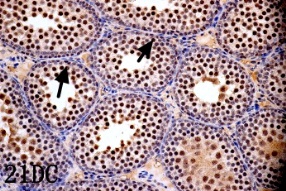 | 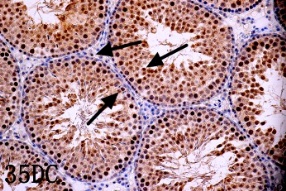 | 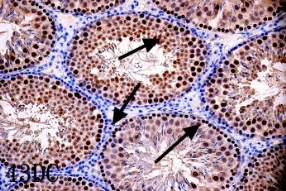 | 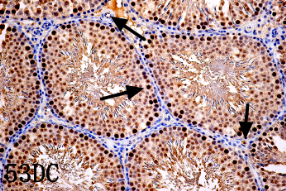 |
| CE | 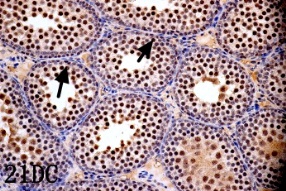 | 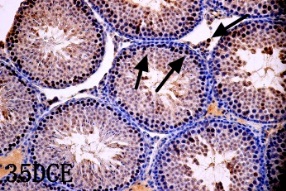 | 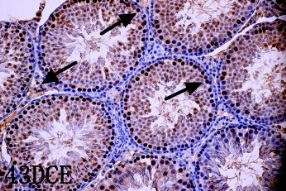 | 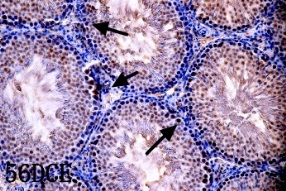 |
| HC | 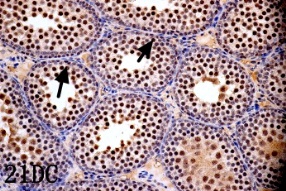 | 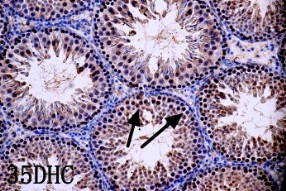 | 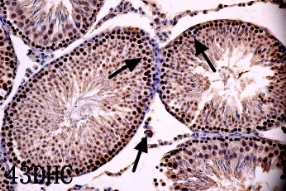 | 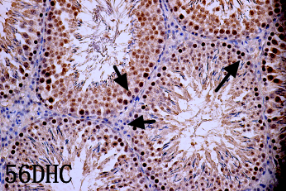 |
| HE | 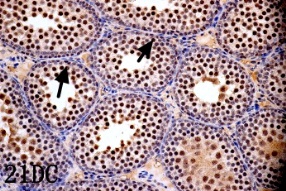 | 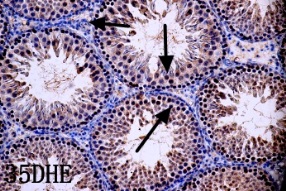 | 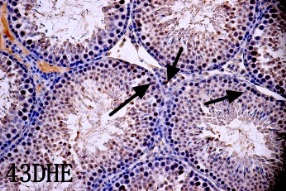 | 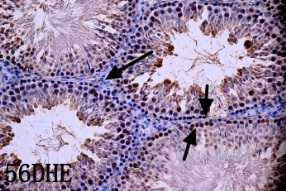 |

（The black arrow indicates the presence of KiSS-1）
